# Evaluation of a new Everolimus-coated balloon catheter in an *in-vivo* porcine peripheral venous model

**DOI:** 10.1186/s42155-025-00530-5

**Published:** 2025-02-27

**Authors:** Stavros Spiliopoulos, Lazaros Reppas, Nikolaos Giannikas, Panagiotis Kitrou, Michail Theofanis, Michail Karpetas, Anargyros N. Moulas, Ioannis Paraskevopoulos, Amalia I. Moula, Kechagias Ioannis, Dimitrios Karnabatidis

**Affiliations:** 1 2nd Department of Radiology, Interventional Radiology Unit, School of Medicine, National and Kapodistrian University of Athens, “Attikon” University General Hospital, Athens, Greece; 2 Department of Interventional Radiology, School of Medicine, Patras University Hospital, Rion, Greece; 3 Anesthesiology and Critical Care Medicine, School of Medicine, University of Patras, 26504 Rion-Patras, Greece; 4 General Department, University of Thessaly, Larissa, Greece; 5 Radiology Department, School of Medicine, University of Ioannina, Ioannina University Hospital, Ioannina, Greece; 6 Department of Surgery, “Achillopouleion” General Hospital, Volos, Greece; 7 Surgery Department, School of Medicine, University of Patras, University Hospital of Patras, Rion, Greece

**Keywords:** Everolimus, Restenosis, Venous system, Angioplasty Drug-coated balloons

## Abstract

**Background:**

The venous uptake following the application of Everolimus-coated balloons is under reported. We evaluated the feasibility, safety, and Everolimus (EVR) deliverability of a novel non-commercially available Everolimus-Coated Balloon (ECB) catheter in a swine healthy peripheral vein model.

**Methods:**

In total 12 ECBs (5.0 μg/mm^2^) were inflated in 12 venous segments. The primary feasibility endpoint was the successful application of the ECB at the target venous sites. The primary efficacy endpoint was the successful drug uptake by the target venous tissue at 24 h and 7 days, assessed by High Performance Liquid Chromatography combined with tandem mass spectrometry. The primary safety endpoint was freedom from major adverse events.

**Results:**

Everolimus was detected in 10 out of 12 (83.33%) tissue samples (all six tissue samples at 24 h post-intervention and in four out of six samples at 7 days). The mean weight of the examined tissue was 0.20604 ± 0.29822 g (range: 0.37475–0.02229 g). The average EVR tissue content detected at 24 h (135.67 ± 204.95 μg/g) was numerically superior, but non-statistically significant to the that detected 7 days post-procedure (96.85 ± 110.89 μg/g). The average quantity of EVR on the balloon after retrieval was 33.9% of the initial drug dose. No adverse events were recorded, and no abnormalities were noted during autopsy.

**Conclusions:**

The newly developed ECB successfully delivered Everolimus within the healthy venous wall. No adverse events were noted at a short-term follow-up.

**Relevance statement:**

These safety and feasibility results justify further experimental and clinical research to demonstrate the safety efficacy the specific balloon catheter.

**Supplementary Information:**

The online version contains supplementary material available at 10.1186/s42155-025-00530-5.

## Key points

-The venous uptake following the application of Everolimus-coated balloons is under reported.

-Safety and drug deliverability data are provided.

-Everolimus was safely and successfully delivered within the healthy venous wall.

**Supplementary Information:**

The online version contains supplementary material available at 10.1186/s42155-025-00530-5.

## Background

Percutaneous transluminal angioplasty (PTA) is the first-line endovascular method for treating benign venous stenosis [1, 2]. However, symptoms relapsing due to restenosis remains the main shortcoming of PTA [3]. Paclitaxel-coated balloons (PCBs) have been recently investigated as a potentially effective alternative for improving patency outcomes and long-term clinical benefit, but results from various prospective studies, including multicenter, randomized trials, provided conflicting evidence [4–10]. 

Sirolimus (rapamycin) and its analogs (the -limus family of cytostatic drugs), has been widely used for the development of drug-eluting stents with proven anti-restenotic effects in the treatment of coronary and peripheral arterial disease [11–13]. Among the advantages of the -limus drugs compared to paclitaxel is the reversible cytostatic mechanism of action (sirolimus is an mTOR inhibitor with potent immunosuppressive properties) which incites cell proliferation cycle arrest at G1/S phase, without triggering cell death, as well as the broader therapeutic window with a 100-fold dosage safety margin, compared to paclitaxel [14]. 

Everolimus could offer several key advantages in venous applications, such as inhibition of smooth muscle cells and fibroblasts, preventing tissue growth without inducing cell death but also reducing the inflammatory response which could result in less venous restenotic lesions due to reduction of macrophage infiltration and slow down intimal thickening.

Until recently, paclitaxel has been the only drug that could be loaded to a balloon coating and to be efficiently delivered within the vessel wall [15]. However, recent technological advancements have enabled the development of -limus-coated balloons, overcoming earlier limitations associated with the drug’s physical properties that rendered the formulation of stable and effective balloon coatings difficult, while preventing an efficient arterial wall uptake, thus resulting in suboptimal treatment efforts [16, 17]. 

Despite extensive preclinical reports on sirolimus-coated balloon application in the ambit of arterial disease, the venous uptake following the application of everolimus-coated balloon angioplasty has not been previously reported in the literature and remains to be determined in order to demonstrate the possibility of its antirestenotic efficacy [18–20]. The proof-of-concept of drug uptake following the application of -limus coated balloons in veins is expected to have a significant clinical impact in the treatment of venous steno-occlusive disease, by inhibiting neointimal hyperplasia and reducing inflammatory responses, which may offers an advantage over traditional angioplasty by prolonging vessel patency and reducing the need for frequent reinterventions. This is particularly relevant in CVS, where short recurrence of stenosis significantly impairs the quality of life in dialysis-dependent patients. The aim of this in-vivo experimental protocol is to evaluate the safety and drug deliverability of a new, non-commercially available, Everolimus-coated balloon (ECB) catheter, in a peripheral vein animal model.

## Materials and methods

Institutional Review Board approval was obtained (approval number: PDE/DK/186321/688) for this experimental protocol which conformed to EU’s directive and EU’s guidelines for the accommodation and care of animals (No. 85–23, rev. in 1996). The non-commercially available over-the-wire, Rontis 5.0 μg/mm^2^ Everolimus-Coated Balloon with a proprietary excipient system (ECB; Rontis Hellas SA, Larissa, Greece), was tested in a healthy swine peripheral vein model, which is the most widely used animal model for the preclinical evaluation of endovascular devices Porcine models are widely used for testing different technologies in the veins and arteries of the lower limbs due to their remarkable anatomical and physiological similarities to humans. In addition, the vascular system along with the response of porcine vessels to injury and drug delivery closely mimics human physiology, allowing to evaluate the safety and efficacy of DEBs [21]. 

The protocol included the application of 6 ECBs in 6 venous segments in each animal.

In total, 2 female swine (mean age 4 months and mean weight 55 ± 3 kg) and 12 ECBs in 12 venous segments were used as follows: common iliac vein; CIV, external iliac vein; EIV and internal iliac vein; IIV, bilaterally). Procedures were performed under general anesthesia (GA) using a straight laryngoscope and 5.5–7.5 mm mouth-tracheal access. A pre-anesthetic bolus dose of intramuscular ketamine (15 mg/kg) and xylazine (2 mg/kg) was administrated followed by anesthetic suppression (propofol 1% at 1–1.4 mg/kg via an ear vein). Vital signs were continuously monitored until recovery.

### Procedure

The procedures were performed in a licensed animal laboratory using a dedicated C-arm equipped with digital subtraction angiography (DSA) capabilities. Following general anesthesia, and aseptic preparation, venous access was obtained via the internal jugular vein using ultrasound-guidance and a micro puncture kit (Merit S-MAK™, Merit Medical, Utah, USA), followed by the placement of a 4Fr × 10 cm sheath over a standard 0.035″ angled hydrophilic wire (Terumo, Europe NV) positioned within the inferior vena cava (IVC). An intra-venous bolus of heparin (100U/kg) was administered, and the jugular sheath was upsized with a 7Fr 90 cm sheath (Terumo, Europe NV), which was advanced to the distal IVC as to perform DSA with 10 mL of 50% contrast/saline mixture (Visipaque, Iodixanol, General Electric, UK) and obtain roadmap for the selective catheterization of the target vessels. In order to simulate standard clinical practice, pre-dilatation was performed using standard semi compliant balloon catheters dilated for 30 s at nominal pressure of 6 bar, followed by the application of the ECB (120 s at 10 bar). The nominal diameters of balloons (pre-dilation and ECBs) expanded in each vein are the following:

- Right common iliac – left common iliac vein (Ø 8 mm).
- Right external iliac – left external iliac vein (Ø 7 mm).
- Right internal iliac – left internal iliac vein (Ø 6 mm).

The above mentioned venous sites were selected to simulate clinical practice, based on their diameter matching the available balloons and the ease of surgical extraction compared to other anatomical locations. Balloon and deployment details, including transit times, are reported in detail in Table 1. After balloon removal, DSA was performed to evaluate and record treated vessel condition (stenosis, dissection, thrombosis, spasm). Hemostasis was achieved by manual compression for 5–10 min. Upon completion of the procedure, the animals were transferred to the recovery room and clopidogrel 75 mg and aspirin 100 mg was administrated once daily, per os from day 1 until the day of euthanasia. Blood samples were drawn at the end of the procedure and just prior to euthanasia. According to the protocol, animal #1 was sacrificed at 24 h (acute) and animal #2 at 7 days using Sodium Pentobarbital. Euthanasia at 24 h and 7 days was performed because the main investigation point was to access the drug pharmacokinetics in the tissues in short time. It is known that pigs age at a rate of 6 years to every human year, so 7 days are approximately a month in human age time. All treated venous segments were harvested following abdominal incision performed by a vascular surgeon with previous experience in swine vascular models. Both tissue and blood samples were placed in appropriately labelled tubes, transported to the analytical laboratory in dry ice, and kept at -80°C until analysis. No specific harvesting protocol was followed. The vascular surgeon used standard vascular surgery techniques and anatomical landmarks, based on the DSA, in order to achieve higher precision.

**Table 1:** Balloon and deployment details. CIV = common iliac vein, EIV = external iliac vein, IIV = internal iliac vein

Vein	Animal	Catheter size (mm)	Transition Time (sec)
LCIV	1	8.0 × 60	23
	2	8.0 × 60	22
LEIV	1	7.0 × 60	27
	2	7.0 × 60	21
LIIV	1	7.0 × 60	25
	2	7.0 × 60	27
RCIV	1	8.0 × 60	25
	2	8.0 × 60	22
REIV	1	7.0 × 60	21
	2	7.0 × 60	19
RIIV	1	7.0 × 60	29
	2	7.0 × 60	19
		**Min**	**19**
		**Max**	**29**
		**Average**	**23.3**
		**St. Deviation**	**3.26**

### Determination of everolimus in tissue, serum and ECBs

Everolimus tissue and serum concentration was determined using high performance liquid chromatography (HPLC) coupled with tandem mass spectrometry (MS/MS) following extraction of everolimus from the sample. Everolimus D4 was used as internal standard for determination of everolimus during the extraction process in serum and tissue samples. Analytical standard of everolimus was purchased from European Pharmacopoeia.

Tissue extraction was performed as follows: A quantity of tissue between 0.005 and 0.050g was weighted with an accuracy of 0.01 mg and transferred to a special cryotube. Liquid nitrogen was added, and the tissue was pulverized with the use of a special tool. The tool was washed with a total of predefined quantity of 990 μL of ethanol and the ethanol solution was collected with the sample and 10 μL of the internal standard solution (20 μg/mL Everolimus D4 in methanol) was introduced. Subsequently, the solution was mixed and sonicated for 15 min at an ultrasonic water bath under temperature control and filtrated using a 0.2μm syringe filter. An aliquot of the extract was diluted 5 × with methanol and 5μL of the diluted extract was loaded to the HPLC system for quantification.

Extraction of everolimus from blood (serum) sample was performed as follows: A total of 10 μL of an internal standard solution (10μg/ml of everolimus D_4_ in methanol) was added in 100μL serum. Serum was deproteinized with a 4 × volume of methanol. After vortexing for 2 min, the deproteinized serum was centrifugated, the supernatant was filtered with a 0.2μm syringe filter and a 5μL volume was loaded to the HPLC system.

Extraction from the balloon catheter was performed as follows: balloons were immersed in 10 mL of absolute ethanol in glass with stopper, left to extract for 48 h and then sonicated for 15 min. A 5.0 μL aliquot of the extract was diluted 200 × with methanol and 5μL of the diluted extract was loaded in the HPLC system.

### Chromatography and MS/MS detection

**Fig. 1:**
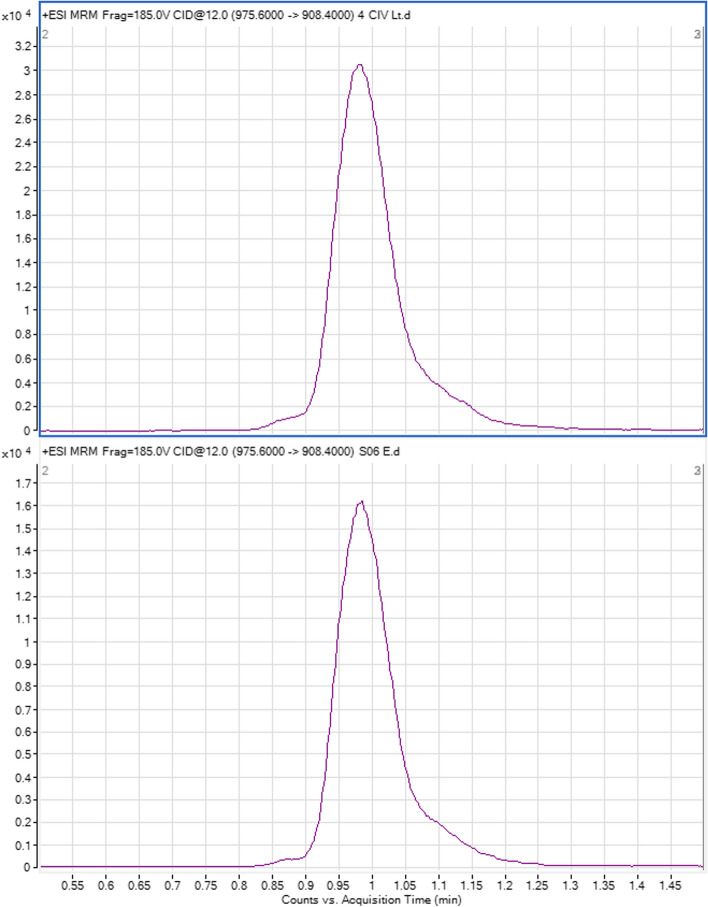
High performance liquid chromatography / tandem mass spectrometry chromatogram of a tissue sample (up) and an everolimus standard (down). The peak at 0.97 min is everolimus. The mass transition shown is the breakdown of the ammonium adduct of everolimus [M + NH4] + 975.6 (m/z) to a high mass fragment of everolimus 908.4 (m/z)

The detection and quantification of everolimus was performed using an Agilent 6430 HPLC/MS/MS system consisting of a degasser model G4225A, a binary pump model G1312B, an autosampler model G1329b, a thermostated column compartment model G1316A, a diode array detector model G4212B, a 35900E interface and an Agilent triple quadrupole mass spectrometer model G6430. System control and data analysis were performed with the use of the Agilent Mass-Hunter QQQ control console and qualitative and quantitative data analysis software programs. The separation was achieved using an Agilent column (type Poroshell 120 EC-C18 3.0 × 50 mm, 2.7 Micron), isocratic elution with a mixture of 85:15(v/v) methanol: water, containing 10mM ammonium acetate and 0.2% formic acid. Standard curves were plotted with the use of known concentrations of analytical standards of everolimus and the internal standards everolimus D_4_ (Fig. 1).

### Study endpoints and definitions

Primary endpoints were feasibility, safety and efficacy of the ECB device. Feasibility was defined as successful application of the devices to the predefined target veins of the animals. Safety was defined as freedom from any major adverse event (death, major cardiovascular event) correlated to the device under investigation during the test period of 24 h and 7 days. A complete autopsy was performed in both animals following euthanasia. Efficacy was defined as successful drug absorbance by the target tissue, following the application of the ECB, in terms of drug uptake levels, assessed by HPLC/mass spectrometry. Secondary endpoints were the investigation of predetermined factors (everolimus concentration on medical device after application and ECB transition time) affecting the primary endpoints and the determination of everolimus in peripheral blood samples 7 days after the application of the ECBs.

### Statistical analysis

Discrete variables were reported as counts / percentages. Continuous variables were expressed as means ± standard deviation (SD). Following Kolmogorov–Smirnov normality testing, the independent samples t-test was used to compare everolimus concentrations in tissue and blood samples between 24h and seven days. Multiple linear regression analysis was performed to perceive a possible correlation between multiple predetermined independent variables [ECB transition time, everolimus remaining on the balloon and time of analysis (24h/7 days)] and the primary outcome of tissue concentration of everolimus. The aforementioned statistical analysis was performed using the SPSS statistical software (version 25.0; IBM, USA).

## Results

### Feasibility and safety

All 12 ECB’s were applied at the target veins following bare balloon pre-dilatation, as per protocol, without complications or major adverse events (death or animal paralysis). The mean transition time was 23.3 ± 3.26 s (range: 19 – 29 s) (Table 1). At the end of the process, post procedural angiographic assessment did not detect any abnormalities of the treated vessel (no spasm, rupture, dissection or stenosis). Both test animals survived until the end of the protocol, i.e., completed 24 h and 7 days without showing any health issues. Macroscopic evaluation of animal health, including the inspection of vital signs, performed by the veterinarian, did not yield any suspicious findings. Following autopsy, there were no abnormalities noted with the exemption of those observed in the muscle tissue attributed to target vessel harvesting. The findings of the autopsy report are analytically reported in Supplementary Table S1. The two serum samples were successfully analyzed for everolimus content. The drug was not detectable in any of the samples. The lack of detectable systemic effects has important implications for the overall safety profile of Everolimus.

### Everolimus quantification

**Fig. 2:**
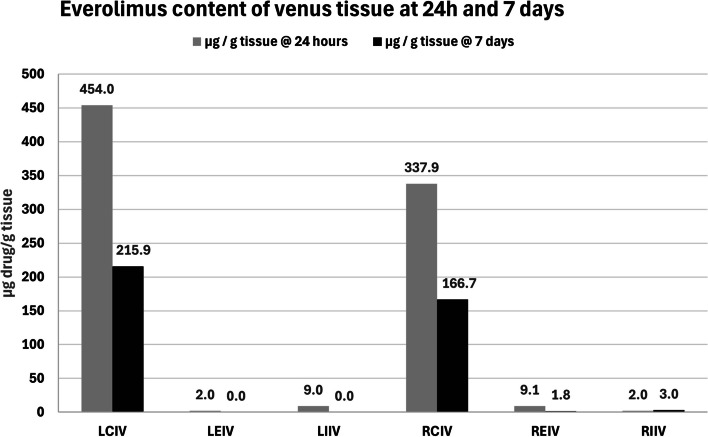
Everolimus detected within venous tissue at 24 h and 7 days. 0,0: non detectable. CIV = common iliac vein, EIV = external iliac vein, IIV = internal iliac vein, L = left, R = Right

Everolimus concentration of the treated vein tissues was measured with HPLC/MS/MS. The mean venous tissue weight analyzed 0.20604 ± 0.29822g (range: 0,0.37475–0,0.02229g) (Table 2). Everolimus was detected in every tissue sample 24h post-intervention (6/6) and in 4/6 tissues 7 days post-intervention (10 tissue samples out of 12 overall). The average everolimus content present in the target venous tissues 24 h post-operation was 135.67 μg/g (range 2 -454) and was higher than the drug quantity measured at day 7 which was 96.85 μg/g (range 1.8 – 215.9), but this difference (mean difference 38.82 μg/g; two-sided p = 0.32) was not statistically significant. Results are analytically reported in Table 2 and demonstrated in Fig. 2. According to statistical analysis, everolimus concentration in the target venous samples was not correlated to the transition time, time of analysis (24h/7 days), or everolimus quantity which remained on the balloon following deployment (Supplementary Table S2). Furthermore, no immediate vascular trauma was noted, while everolimus was not detectable in peripheral blood at 24h and 7 days, implicating that non-targeted systemic action should not be expected, although this remains to be determined by long-term protocols with non-target tissue examination of distal limb muscles, as well as pulmonary and cardiac tissue, to address possible safety issues created by the properties of the medium of delivery and the longer the tissue half-life of everolimus possibly due to poorer solubility in comparison to Paclitaxel [20].

**Table 2:** Everolimus detected within venous tissue at 24 h and 7 days

Artery	Animal	Coated Drug	Weight of tissue (g)	μg / g tissue @ 24 h	μg / g tissue @ 7 days
LCIV	1	Everolimus	0.28941	**-**	**215.9**
	2		0.35010	**454.0**	**-**
LEIV	1		0.14351	**-**	**N.D**
	2		0.10913	**2.0**	**-**
LIIV	1		0.02229	**-**	**N.D**
	2		0.05446	**9.0**	**-**
RCIV	1		0.37475	**-**	**166.7**
	2		0.17175	**337.9**	**-**
REIV	1		0.17451	**-**	**1.8**
	2		0.06891	**9.1**	**-**
RIIV	1		0.05129	**-**	**3.0**
	2		0.05986	**2.0**	**-**
			**Min**	**2.0**	**1.8**
			**Max**	**454.0**	**215.9**
			**Average**	**135.67**	**96.85**
			**St. Deviation**	**204.954**	**110.897**

### Everolimus quantity remaining on the balloon catheter post-intervention

The measurements of drug content on the surface of the balloons following deployment and retraction provided by the analytical laboratory demonstrated that an average of 33.9% of the everolimus drug remains on ECBs (range of measurements was between 12 – 53%), therefore an average of 66.1% of the original drug load is partly washed off during balloon advancement through the introducer sheath, during tracking towards the lesion, during balloon inflation and partly uptaken into the vascular endothelium at the target vascular site. The remaining drug quantity on each balloon is analytically reported in Table 3.

**Table 3:** Everolimus content per balloon (remaining post-intervention). CIV = common iliac vein, EIV = external iliac vein, IIV = internal iliac vein, L = left, R = Right

Artery	Animal	Everolimus found (μg)	Initial coating weight (μg)	Initial drug content (μg)	Remained quantity as % of initial content
LCIV	1	**2148**	12,700	7697	**28**
	2	**2148**	12,700	7697	**28**
LEIV	1	**3210**	11,595	7027	**46**
	2	**812**	10,930	6624	**12**
LIIV	1	**3138**	9825	5955	**53**
	2	**1816**	10,770	6527	**28**
RCIV	1	**2834**	11,775	7136	**40**
	2	**2450**	12,785	7748	**32**
REIV	1	**1809**	10,585	6415	**28**
	2	**2510**	10,515	6373	**39**
RIIV	1	**1463**	10,740	6509	**22**
	2	**3094**	9945	6027	**51**
				**Min**	**12**
				**Max**	**53**
				**Average**	**33.9**
				**St. Deviation**	**0.12**

## Discussion

According to the results of this experimental protocol this novel ECB successfully delivered a substantial dose of everolimus within the venous wall of the majority of the samples, although a significant variability of drug amount was noted in both timeframes. Most importantly, the dose of everolimus detected 7 days following ECB dilation was similar to that detected at 24 h, demonstrating the possibility of a mid-term antirestenotic efficacy of the balloon catheter. Moreover, the mid-term safety of the ECB under investigation was demonstrated by the absence of adverse events during the 1-week study period, further confirmed by autopsy. The above safety and drug-deliverability proof-of-concept findings could be used support the clinical application of the specific ECB catheter in patients with venous steno-occlusive disease referred for endovascular treatment in specialized vascular centers.

With respect to the comparison of the drug quantities detected in previous experimental studies investigating the drug uptake of a paclitaxel-coated balloon catheter in the same swine venous model, the uptake of Everolimus was, evidently, much higher than that of Paclitaxel in both timeframes [22]. Nevertheless, substantial variability in drug uptake within the vessel wall was observed (Table 2). This may be due to harvesting errors or variations in venous diameter, even within the same vein, between the proximal (larger) and distal (smaller) segments, affecting balloon apposition and possibly drug deliverability. Moreover, variability in tissue weights, may have also influenced drug concentration measurements. It should be noted that such variability of drug uptake could influence the clinical efficacy of the balloon catheter, in cases of very low drug uptake, below the therapeutic threshold.It is difficult to interpret and discuss the absence of drug detected in two tissue samples 7 days post-procedure because of the very high deviation in drug quantities detected in the tissues corresponding to either timeframe. It could probably be attributed to erroneous harvesting, as the precise boundaries of venous endothelium engaged by the inflated ECBs was determined by anatomical markers only. This hinders the validity of any conclusions drawn as to the rate of local drug metabolism in 7 days, as well as the physiological importance of comparing the average drug detected in the two timeframes, despite the fact that results correlated with the reasonable assumption of expecting a lower average quantity to be associated with a longer time period post-procedure. The use of a commercially available ECB catheter would have also provided another control in measuring and comparing the level of drug lost or absorbed into the tissue per group per timeframe. Considering the small sample size discussed in this study, the application of a paclitaxel-coated balloon catheter in the same swine model, was associated with a much lower deviation of drug detected in the tissue samples, than that observed in the present protocol according to previously reported experimental data [22]. Additionally, in comparison, the paclitaxel coated balloon has been shown to retain an average of 33.8% (range of measurements varying between 20—44), indicating a very similar level of average drug loss at 66.2% [22]. 

The limitations of this experimental study include the absence of a power analysis and the relatively small number of veins analyzed. These limitations restrict the scope of robust statistical analysis, and consequently, all results, particularly those concerning drug uptake between 24 h and 7 days, should be interpreted with caution. Further limitations include the absence of a control group with a commercially available -limus coated or paclitaxel- coated catheter, the absence of a negative control venous tissue where no ECB has been deployed and the lack of sampling from peripheral organs in order to investigate possible systemic effects due to drug wash-off. Thus, larger studies incorporating both healthy and pathological vein samples are required to ensure positive clinical results. Moreover, the timepoints examined in this protocol did not allow the process of intimal hyperplasia triggered by bare balloon pre-dilation to form clinically applicable lesions that could provide information on the drug (Everolimus) effect in non-healthy, clinically-relevant vascular environment. In contrast, the in vivo model employed was a healthy peripheral venous swine model, therefore data on drug deliverability and absorption in stenotic veins, as to simulate clinical scenarios, are not provided. Finally, data regarding the long-term patency and efficacy of the specific ECB were not available due to the short timeline of the study, but also due to the absence of a control group undergoing bare balloon angioplasty on the contralateral vein. Patency outcomes remain to be investigated by future studiesIn this study, short-term dual antiplatelet therapy (clopidogrel/aspirin) was administrated based on evidence deriving from arterial interventions [23]. However, this could create a bias, as dual antiplatelet therapy is not routinely recommended following venous interventions.

In conclusion, these results indicate that application of the novel ECB in a peripheral venous model is feasible without any specific safety issue.

## Supplementary Information

Supplementary Material 1:  Supplementary Table S1. Autopsy report.

Supplementary Material 2:  Supplementary Table S2. Multiple linear regression analysis outcomes.

## Data Availability

The datasets generated and/or analyzed during the current study are not publicly available due to commercial reasons, but are available from the corresponding author on reasonable request.

## Abbreviations

CIV: Common Iliac Vein
CVS: Central Venous Stenosis
DSA: Digital Subtraction Angiography
ECB: Everolimus-coated balloon catheter
EIV: External Iliac Vein
EVR: Everolimus
GA: General Anaesthesia
IIV: Internal Iliac Vein
PCB: Paclitaxel-coated balloon catheter
mM: Millimolar
