# Supplementary material for: Evaluation of a new Everolimus-coated balloon catheter in an in-vivo porcine peripheral venous model
Source: CVIR Endovasc. 2025 Feb 27;8:16. doi: 10.1186/s42155-025-00530-5 (PMC11867998; doi:10.1186/s42155-025-00530-5)
Supplement: Supplementary file 1 — Supplementary Material 1: Supplementary Table S1. Autopsy report. [file 42155_2025_530_MOESM1_ESM.docx]

| **Skull** |  |  |
| --- | --- | --- |
| Cranial cavity | - Cerebrospinal fluid - Brain, cerebellum | Normal  Normal |
| Facial skull | - Cross section and observation of nasal cavities - Oral cavity - Pharynx | Normal  Normal  Normal |
| **Thorax** |  |  |
| Respiratory system | - Lungs, Pulmonary parenchyma - Trachea, bronchi - Pleura, diaphragm | Normal  Normal  Normal |
| Circulatory system | - Heart, Myocardium, Pericardium - Endocardium - Aortic arch | Normal  Normal  Normal |
| **Abdomen** |  |  |
| Peptic system | - Peritoneum, peritoneal fluid - Stomach - Small intestine, Cecum - Large intestine | Normal  Normal  Normal  Normal |
| Urogenital system | - Kidneys, renal parenchyma, calyces - Bladder | Normal  Normal |
|  | - Uterus, Ovaries - External genitals | Normal  Normal |
| **Muscle tissue** | - Multiple incisions at the iliac and femoral region | Normal |
| **Skeletal system** | - Bones, cartilage - Intra-articular fluid – Articular surfaces | Normal  Normal |
