# Supplementary material for: Evaluation of a new Everolimus-coated balloon catheter in an in-vivo porcine peripheral venous model
Source: CVIR Endovasc. 2025 Feb 27;8:16. doi: 10.1186/s42155-025-00530-5 (PMC11867998; doi:10.1186/s42155-025-00530-5)
Supplement: Supplementary file 2 — Supplementary Material 2: Supplementary Table S2. Multiple linear regression analysis outcomes. [file 42155_2025_530_MOESM2_ESM.docx]

**Table 4.** Multiple linear regression analysis outcomes

| **Model Summary^b^** | | | | |
| --- | --- | --- | --- | --- |
| Model | R | R Square | Adjusted R Square | Std. Error of the Estimate |
| 1 | .534^a^ | .285 | .016 | 12.96807130 |
| a. Predictors: (Constant), TransitionTime, RetainedDrug, Day | | | | |
| b. Dependent Variable: Concetration | | | | |

| **ANOVA^a^** | | | | | | |
| --- | --- | --- | --- | --- | --- | --- |
| Model | | Sum of Squares | df | Mean Square | F | Sig. |
| 1 | Regression | 535.430 | 3 | 178.477 | 1.061 | .418^b^ |
|  | Residual | 1345.367 | 8 | 168.171 |  |  |
|  | Total | 1880.797 | 11 |  |  |  |
| a. Dependent Variable: Concetration | | | | | | |
| b. Predictors: (Constant), TransitionTime, RetainedDrug, Day | | | | | | |

| **Coefficients^a^** | | | | | | | | | | | | | |
| --- | --- | --- | --- | --- | --- | --- | --- | --- | --- | --- | --- | --- | --- |
| Model | | Unstandardized Coefficients | | Standardized Coefficients | t | Sig. | 95,0% Confidence Interval for B | | Correlations | | | Collinearity Statistics | |
|  |  | B | Std. Error | Beta |  |  | Lower Bound | Upper Bound | Zero-order | Partial | Part | Tolerance | VIF |
| 1 | (Constant) | 47.100 | 27.451 |  | 1.716 | .125 | -16.202 | 110.402 |  |  |  |  |  |
|  | Day | -11.930 | 7.920 | -.476 | -1.506 | .170 | -30.193 | 6.334 | -.469 | -.470 | -.450 | .894 | 1.119 |
|  | RetainedDrug | -.234 | .595 | -.124 | -.393 | .705 | -1.605 | 1.138 | -.269 | -.138 | -.117 | .902 | 1.108 |
|  | TransitionTime | -.468 | .710 | -.205 | -.659 | .528 | -2.104 | 1.169 | -.137 | -.227 | -.197 | .925 | 1.081 |
| a. Dependent Variable: Concetration | | | | | | | | | | | | | |
